# Standardizing the Bactericidal Activities of Silver Nanoparticles Made By Electrochemical Reduction and Comparing It with Deconex 53 Instrument

**Published:** 2011

**Authors:** Omid Rajabi, BiBi Sedigheh Fazly Bazzaz, Ali Reza Vaseghi, Roshanak Salari

**Affiliations:** ***Departmant of Medicinal Chemistry, School of Pharmacy, Mashhad University of Medical Sciences (MUMS), Mashhad, Iran.***

**Keywords:** Silver nanoparticles, Deconex, Disinfectant, Standards

## Abstract

Silver nanoparticles have proved to possess significant antibacterial properties. Nanosilver produced by electrochemical reduction stabilized by cellulose derivatives, has been evaluated by European standards (CEN TC 216) in order to find out whether they could be applied in food, industrial, domestic and institutional areas as a suitable disinfectant. Moreover, bactericidal activity of nanosilver has been compared to Deconex 53 Instrument, which is a potent disinfectant. Silver nanoparticles not only passed the standards, but also showed a competitive bactericidal activity against Deconex 53.

## Introduction

Antimicrobial properties of silver nanoparticles have been broadly investigated by several researches throughout the last decade. The main difference among such studies is due to the various synthesis methods applied, yielding different shapes, sizes, optical properties and bactericidal effects (1-5, 11, 12). However, they all agree upon the fact that nanosilver is such a precious target at delivering alternative methods of disinfection as the issue of antibiotic resistance has been raised (4-6, 2, 10).

*Escherichia coli *(6, 8, 10), *Staphylococcus aureus *(3, 10)*, methicillin-resistant Staphylococcus aureus *(11)*, Staphylococcus epidermis *(4) and *Bacillus subtilis *(4) are among the targeted microorganisms used in the studies.

It has been concluded that nanomaterials could prove to be simple, cost effective and suitable for formulation of new type of bacterial materials (6, 7, 11). Therefore, it would be beneficial to set some specific standards through which bactericidal efficacy of silver nanoparticles made by various synthesis methods could be assessed. 

Concerning the ever-growing application of nanosilver in various fields (13-14), we managed to access CEN TC 216 standards relating to the evaluation of chemical disinfectants and antiseptics used in food, industrial, domestic and institutional areas.

## Experimental

*Materials*

*Preparation of silver nanoparticles*

The whole procedure of nanosilver synthesis included the two steps of seeding and growing. Seeding was done by electrochemical reduction of silver salt, followed by growing them in a different medium containing sodium citrate as a reducing agent and hydroxypropyl methylcellulose (HPMC) as a reducing and capping agent at 80°C (1, 2).

*Deconex 53 instrument*

Deconex 53 (Borer chemie) is an aldehyde-free, non-fixing disinfectant for manual disinfection and cleaning of surgical instruments, care utensils and dental instruments. 

Typically, deconex53 instrument is being used for decontamination or pre-treatment of surgical and ward instruments. The product can also be used for final disinfection of non-critical medical devices. Active Ingredients of deconex 53 include Didecyl dimethyl ammonium chloride and N-(3-aminopropyl)-N-dodecylpropane-1, 3-diamine . 

**Figure 1:**
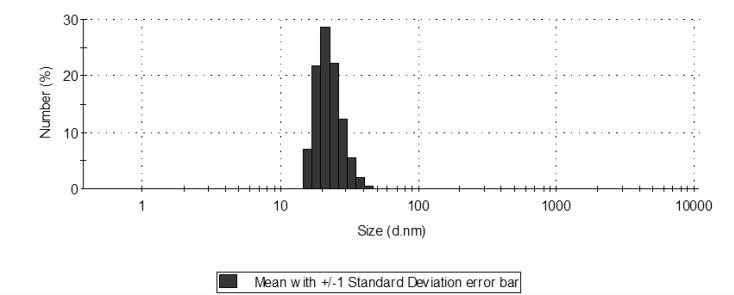
Size distribution of silver nanoparticles with a mean of 22.8 (d.nm).

*Methods*

*Characterization of silver nanoparticles*

The synthesized nanoparticles were characterized by Ultraviolet-visible (UV-visible) spectroscopy (Shimadzu) and further examined by particle-size analyzer [Zetasizer (Nano-zs) by Malvern] to find out its size distribution.

*Bacterial strains and preparation*

Bacterial strains included *Pseudomonas aeruginosa *(ATCC 15442), *Staphylococcus aureus *(ATCC 6538) and *Escherichia coli *(ATCC 10536). The 24 h cultured colonies were used to yield 10^7^ CFU/mL of each bacterial strain in normal saline. Plate counts of 10^-4^, 10^-5^ and 10^-6^ dilutions were done to confirm the initial bacterial load. 

*Bacterial susceptibility*

The antibacterial potency of the synthesized silver nanoparticles was analyzed according to the CEN TC 216. Available standards were: (a) EN 1040:1997 (phase 1) which is a quantitative suspension test for the evaluation of basic bactericidal activity of chemical disinfectants and antiseptics; (b) EN 1276: 1997 (phase 2 step 1) which evaluates the bactericidal activity of chemical disinfectants and antiseptics used in food, industrial, domestic and institutional areas; (c) WI 216028: CEN Enquiry 1999 (phase 2 step 2) which evaluates bactericidal activity on surfaces of chemical disinfectants and antiseptics used in food, industrial, domestic and institutional areas (18).

Four dilutions of silver nanoparticles and Deconex 53 were made (geometric range two) through distilled water for phase 1 and hard water for phase 2. Standards indicate that dilutions should be prepared in such a way that include at least one in the active and one in the inactive range (two in the active range for phase 1). Silver nanoparticles were exposed to bacterial inoculums for 5, 15, 30 and 60 min at 20°C in phase 1 and 5 min for phase 2. In phase 2, the influence of interfering substances including bovine serum albumin (0.3% for 1^st ^step and 3% for the 2^nd^ step), skimmed milk (Himedia) and yeast extract (Fluka) in the efficacy of the disinfectants, was assessed (dirty condition). Survivor count was done by inoculation in the tumor-specific antigen (TSA) culture and incubation at 37°C for 48 h.

## Results and Discussion

UV-visible spectroscopy revealed surface plasmon absorption with two peaks at 322 and 450 nm. According to the Mie theory (19), only a single Surface plasmon resonance (SPR) band is expected in the absorption spectra of spherical nanoparticles, whereas, anisotropic particles could give rise to two or more SPR bands depending on the shape of the particles. The number of SPR peaks increases as the symmetry of the nanoparticle decreases.

Size distribution showed a range of 15.69 to 50.75 with a mean of 22.8 nm. Of course, smaller nanoparticles show more antibacterial properties due to the appropriate penetration through microorganisms’ membrane (2).

*Phase 1*

**Figure 2:**
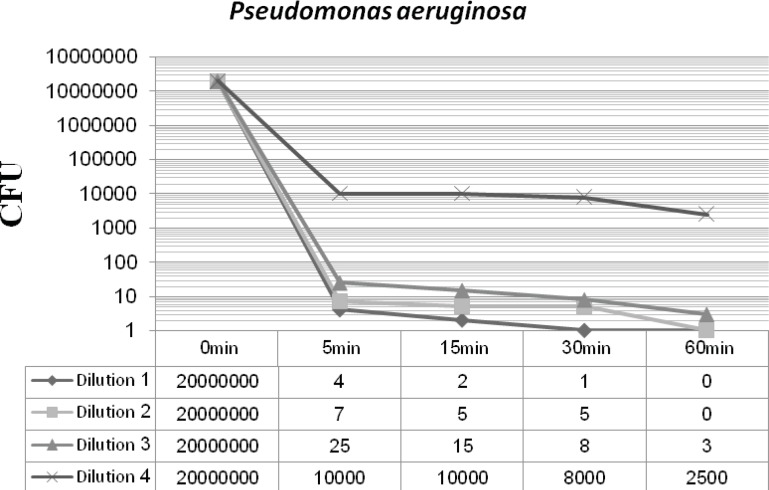
*Staphylococcus aureus*-nanosilver survivor count after 48 h incubation at 37°C (phase 1).

**Figure 3:**
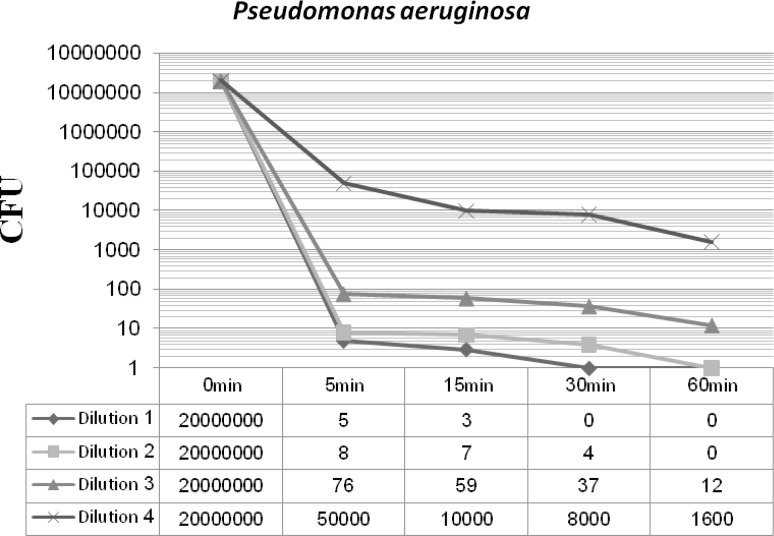
*Pseudomonas aeruginosa *-nanosilver survivor count after 48 h incubation at 37°C (phase 1).

Figures 2 and 3 display the antibacterial potency of four dilutions of synthesized nanosilver against *Staphylococcus aureus *and *Pseudomonas aeruginosa *in phase 1. The first three dilutions showed a significant bactericidal effect for both of the tested strains (6 to 7 log-reduction of the initial inoculums), while the fourth dilution only reduced the initial bacterial load by 3 to 4 log-reduction. According to the EN 1040 (phase 1) standard, 5-log reduction in viability within 60 min or less with both microorganisms, is essential. So this formulation could successfully pass this phase.

Deconex 53 Instrument showed a significant bactericidal effect by inhibiting the growth of both strains in all four dilutions (0.25, 0.5, 1 and 2%) at the tested times.

*Phase 2, step 1*

**Figure 4:**
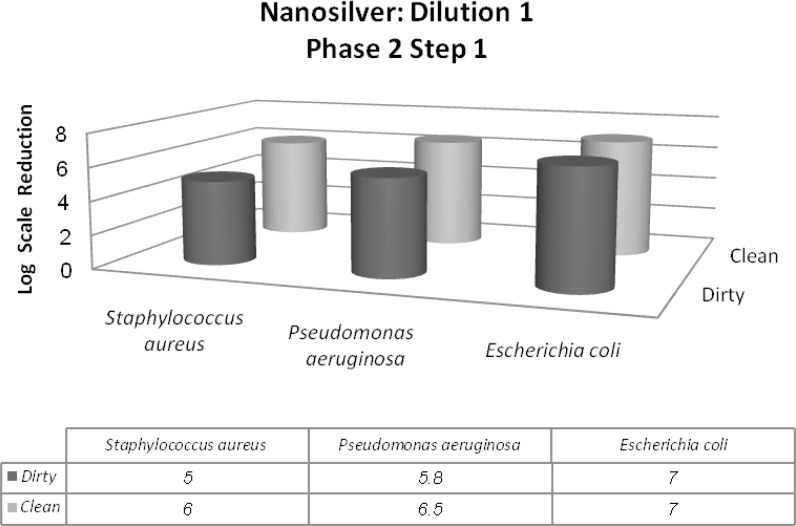
Reduction in the 107 inoculum for the three bacterial strains after exposure to the first dilution of nanosilver for 5 min followed by 48 h incubation at 37°C (phase 2, step 1).

**Figure 5:**
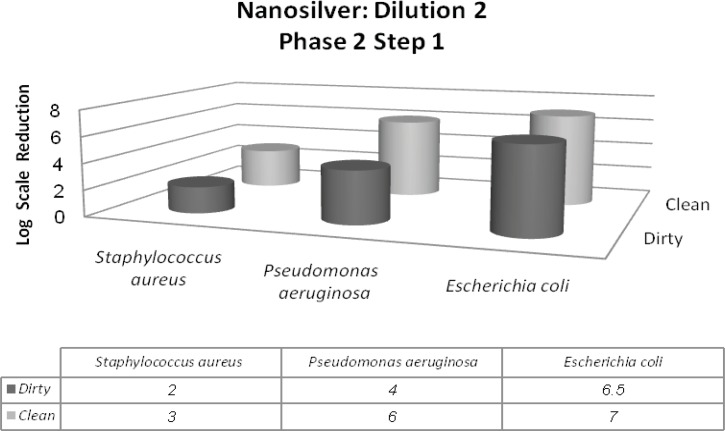
Reduction in the 107 inoculum for the three bacterial strains after exposure to the second dilution of nanosilver for 5 min followed by 48 h incubation at 37°C (phase 2, step 1).

In this phase, two different conditions were considered dirty and clean. It is due to simulating the tested conditions to industrial, domestic and institutional areas. Materials such as bovine serum albumin (BSA), yeast extract and skimmed milk were used for this purpose. In addition to *Staphylococcus aureus *and *Pseudomonas aeruginosa*, *Escherichia coli *was introduced in this phase. The 1^st^ dilution of nanosilver passes the 1^st^ step of 2^nd^ phase with the minimum of 5-log reduction of Staphylococcus aureus and maximum of 7-log reduction of Escherichia coli. On the other hand, the 2^nd^ dilution was in the inactive range (Figures 4 and 5).

**Figure 6:**
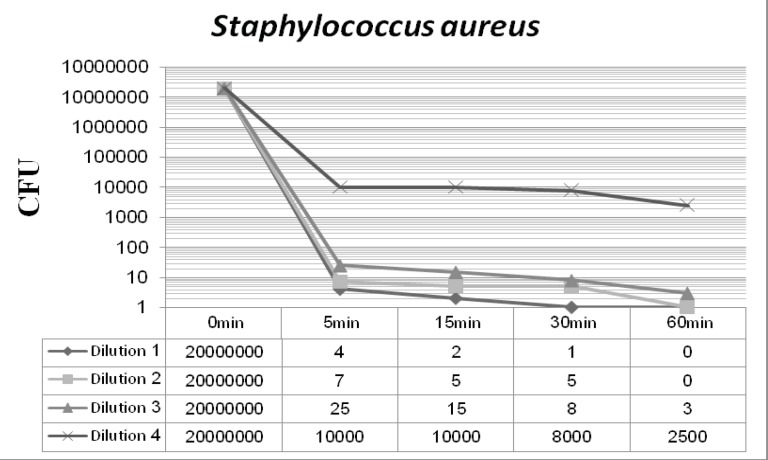
Reduction in the 107 inoculum of *Staphylococcus aureus *after exposure to the 0.1, 0.05 and 0.025% dilutions of Deconex 53 for 5 min followed by 48 h incubation at 37°C (phase 2, step 1).

Deconex 53 was diluted to reach the inactive range. Figure 6 shows the 0.1, 0.05 and 0.025% dilutions and their effectiveness in the dirty condition, by which 0.25% dilution was in the inactive range and 0.1 and 0.05% dilutions passed the 1^st^ step of 2^nd^ phase by minimum reduction of *Staphylococcus aureus *with 5 units in logarithmic scale.

*Phase 2, step 2*

**Figure 7:**
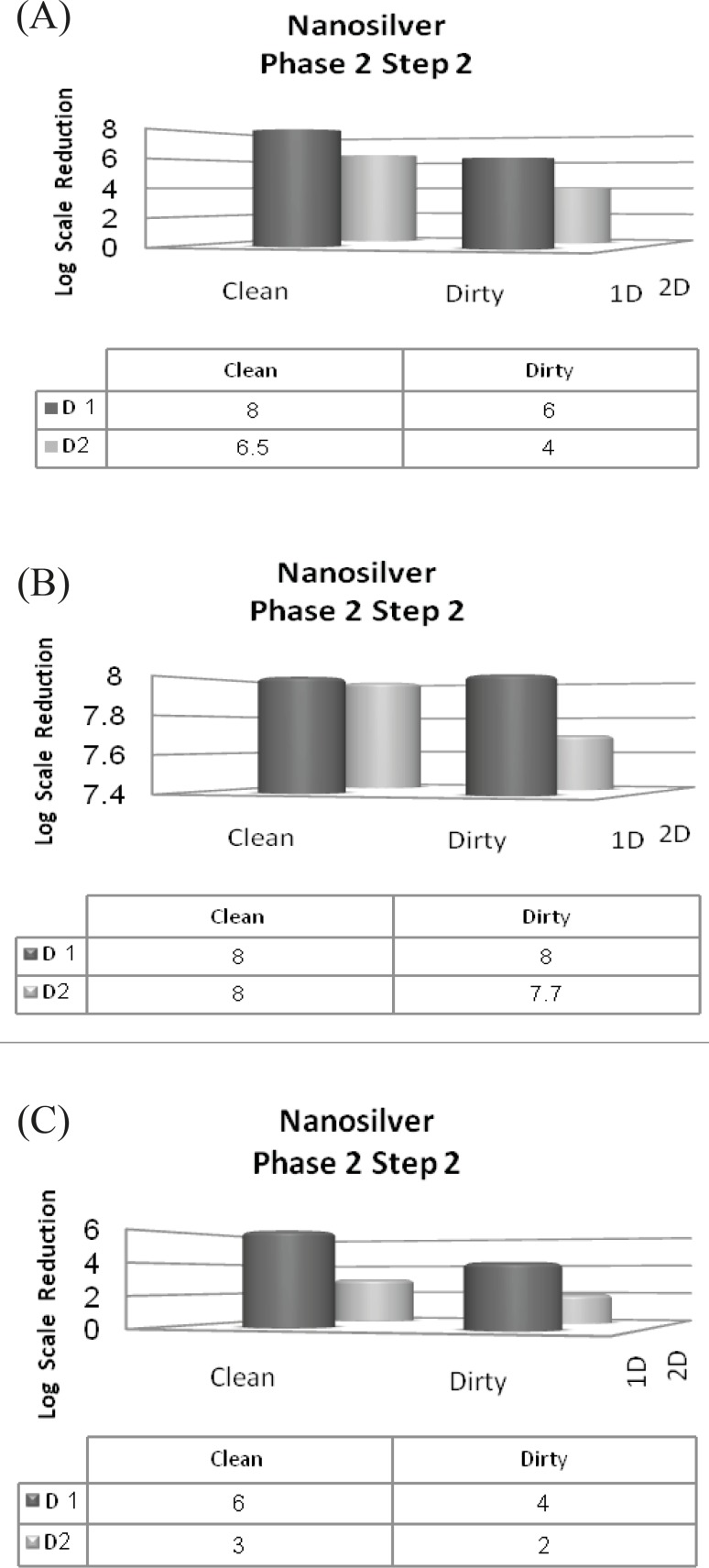
Reduction in the 10^8^ inoculum of the three standard strains after exposure to the two dilutions of nanosilver in clean and dirty conditions (phase 2, step 2) (A) *Pseudomonas aeruginosa*; (B) *Escherichia coli; *(C) *Staphylococcus aureus*

In spite of previous steps that 10^7^ inoculums were used for all strains, in this step, we should have used 10^8^ inoculums. Two different conditions were also prepared in this step. A 4-log reduction in viability is necessary to pass this step. Only the first dilution of nanosilver passed the 2^nd^ step of phase 2, by displaying a minimum 4-log reduction for *Staphylococcus aureus *(Figure 7-C) yet inhibiting the growth of *Escherichia coli *in both clean and dirty conditions (Figure 7-B). It could be understood that interfering agents could not affect Escherichia coli susceptibility because of its high sensitivity to silver nanoparticles. Unfortunately, second dilution caused less than 4-log reduction in viability of *Staphylococcus aureus*. Hence, second dilution could not pass this step.

From 0.1 and 0.05% dilutions of Deconex 53 that passed the step 1, only 0.1% dilution could pass the 2^nd^ step as well by reducing the initial inoculum by the minimum of 4-log scale .

## Conclusion

It was observed that among the three standard tested organisms, *Escherichia coli *showed the highest sensitivity to silver nanoparticles, which supports the results of another study in this topic (6). On the other hand, *Staphylococcus aureus *was the most stubborn among the three strains. The presence of interfering substances such as albumin was important in the efficacy of the bactericidal properties of nanosilver; it could be said that the more the concentration of interfering substances, the higher the concentration of the nanosilver should be to provide the same bactericidal effect.

Silver nanoparticles produced in this study, proved to have a prominent bactericidal activity and compared to Deconex 53, as a strong chemical disinfectant, are competitive disinfectants.

Further standards could be applied to qualify nanosilver for specific uses in other areas such as medical and veterinary. Moreover, fungicidal and sporicidal activity of silver nanoparticles could be analyzed as well.
